# Supplementary material for: Phenotypic stratification predicts the pace, but not the outcome, of continence recovery after radical prostatectomy
Source: PLoS One. 2025 Dec 18;20(12):e0338900. doi: 10.1371/journal.pone.0338900 (PMC12714187; doi:10.1371/journal.pone.0338900)
Supplement: Attachment — Submitted filename: PONE-D-25-33254_Response_to_Reviewers.docx [file pone.0338900.s005.docx]

**PONE-D-25-33254_Response_to_Reviewers**

**Manuscript title:** *Phenotypic Stratification Predicts the Pace, but Not the Outcome, of Continence Recovery after Radical Prostatectomy*
**Journal:** PLOS ONE
**Reviewer:** #1 Ali Hashemi Gheinani
**Academic Editor:** Stanisław Jacek Wroński, M.D., Ph.D., FEBU

**General Response**

We would like to thank the Academic Editor and Reviewer #1 for their thorough and constructive feedback. We carefully addressed all methodological and editorial comments, performed the requested additional statistical analyses, and revised the manuscript accordingly. Below, we provide a point-by-point response. Reviewer comments are shown in *italics*, followed by our responses in **bold**, with corresponding manuscript changes indicated by section and paragraph.

**1. Technical soundness and clustering methodology**

*“The smallest cluster (n=20) raises issues of statistical robustness, and the absence of validation methods such as bootstrapping, resampling, or consensus clustering limits confidence in the stability of this subgroup.”*

**Response:** We have now performed a comprehensive validation of the 3-cluster solution. Internal validation indices (silhouette, Calinski–Harabasz, Davies–Bouldin), bootstrap stability (Jaccard indices, B = 1000), consensus clustering (PAC), and sensitivity analyses (Gaussian Mixture Models, PCA-based k-means; ARI) were applied. Results are summarized in the revised **Table 3** and accompanying text in the *Results* section. Cluster 2 (n=20) showed lower stability (Jaccard 0.722), which is now explicitly acknowledged in both the *Results* and *Discussion* sections, with a cautionary note regarding interpretation. Details of the validation methods were added to the *Statistical Analyses* section.

**Location of changes:**

- Methods: Statistical analyses (paragraph 3)
- Results: Table 3 and new subsection on cluster validation
- Discussion: new methodological paragraph; revised Limitations paragraph (Cluster 2 stability)

**2. Multiple testing correction**

*“Multiple testing: No correction for multiple comparisons was applied. While the authors justify this by calling the study exploratory, it increases the risk of false positives. The fix doesn’t have to be heavy—add a light-touch sensitivity analysis and more transparent reporting.”*

**Response:** We introduced multiple testing correction using the Benjamini–Hochberg procedure within three predefined families of tests (A: baseline characteristics, B: continence outcomes by cluster, C: early vs. late recovery comparisons). This is described in the revised *Statistical Analyses* section. A new **Table 2** summarizes both raw and adjusted p-values. In the *Results* section, interpretations were updated to reflect adjusted significance levels.

**Location of changes:**

- Methods: Statistical analyses (paragraph 4, multiple testing correction added)
- Results: Table 2 and accompanying text

**3. Predictive modeling details**

*“Predictive models: Logistic regression and random forest were mentioned, but details (feature selection, cross-validation strategy, sample split) are insufficient.”*

**Response:** We clarified the modeling strategy (logistic regression and random forest, 5-fold cross-validation repeated 20 times) in the *Statistical Analyses* section. We also emphasized in the *Results* and *Discussion* sections that predictive performance was limited (AUC < 0.65), aligning with the reviewer’s observation.

**Location of changes:**

- Methods: Statistical analyses (final paragraph)
- Results / Discussion: updated text on predictive modeling limitations

**4. Data availability**

*“The authors state that data are not publicly available due to privacy and ethical restrictions but may be shared upon reasonable request. This does not comply with PLOS ONE’s open data policy…”*

**Response:** We have updated the *Data Availability Statement* to comply with PLOS ONE requirements. We explain that individual-level data cannot be publicly deposited due to ethical and legal restrictions, but de-identified data can be shared with qualified researchers upon reasonable request and ethics approval. Summary-level data underlying figures and tables will be uploaded as Supporting Information.

**Location of changes:**

- Manuscript: Data Availability Statement
- Submission form: revised accordingly

**5. Figures and Tables**

**Response:** We updated figure and table numbering to integrate the new Table 2 (multiple testing results) and Table 3 (cluster validation). Figure file names and formats have been adapted to PLOS ONE guidelines and will be processed through the PACE system prior to resubmission.

**6. Discussion updates**

**Response:** The *Discussion* section was expanded to include (1) methodological validation of clustering, (2) interpretation of the lower stability of Cluster 2, (3) the role of multiple testing correction, and (4) limitations of predictive models. The limitations paragraph was revised to explicitly acknowledge Cluster 2 instability.

**Location of changes:**

- Discussion: new paragraph after clinical interpretation section
- Limitations: first paragraph revised

**7. Abstract updates**

**Response:** Minor modifications were made to the *Abstract* to reflect the additional statistical methods (cluster validation and multiple testing correction) and updated numerical results. The *Methods* subsection now specifies the use of internal validation indices and multiple testing correction, while the *Results* subsection includes updated continence rates and the identification of three clusters (n = 76, 86, and 20). These changes ensure that the Abstract is consistent with the revised analyses presented in the main text.

**Location of changes:**

- Abstract: Methods and Results subsections

**8. Minor language and formatting**

**Response:** We proofread the entire manuscript for consistency, grammar, and terminology (e.g., chi-squared vs χ², spacing, reference formatting). Tense and style were aligned with PLOS ONE conventions.

**Summary Table of Major Changes**

| Reviewer Comment | Action Taken | Location |
| --- | --- | --- |
| Cluster validation lacking | Performed internal, bootstrap, consensus, sensitivity analyses | Methods, Results (Table 3), Discussion |
| Multiple testing correction | Added BH procedure, new Table 2 | Methods, Results |
| Predictive modeling details missing | Expanded description, emphasized AUC < 0.65 | Methods, Results, Discussion |
| Data availability | Revised statement, summary data as SI | Data Availability |
| Figures & Tables | Renumbered, prepared for PACE | All |
| Discussion | Expanded for methodological points | Discussion |
| Language | Final proofread | Whole text |

**Prof. Paweł Macek, PhD, MSc**
Corresponding Author
On behalf of all co-authors
*Collegium Medicum, Jan Kochanowski University of Kielce, Poland*
*Scientific Research, Epidemiology and R&D Centre,*
*Holycross Cancer Centre, Kielce, Poland*
[pawel.macek@onkol.kielce.pl](mailto:pawel.macek@onkol.kielce.pl)
