# Supplementary material for: Phenotypic stratification predicts the pace, but not the outcome, of continence recovery after radical prostatectomy
Source: PLoS One. 2025 Dec 18;20(12):e0338900. doi: 10.1371/journal.pone.0338900 (PMC12714187; doi:10.1371/journal.pone.0338900)
Supplement: Attachment — Submitted filename: Response_to_Reviewer_2_Round_3_Pone-D-25-33254.docx [file pone.0338900.s006.docx]

**Response to Reviewer #2**

Dear Reviewer,

We thank you very much for your thoughtful and constructive feedback. We appreciate the opportunity to further improve our manuscript. Below, we provide detailed responses to all comments, accompanied by the corresponding revisions to the text. All changes requested have been incorporated into the manuscript using Track Changes.

**Abstract**

**Comment 1:**
*“In the method section, it is not clear what the clustering is based on. Phenotype? Incontinence or...? Please explain fully.”*

**Response:**
Thank you for this important observation. We have revised the Methods section of the abstract to clarify the basis of the clustering. The sentence now reads:

“K-means clustering was applied to 11 baseline clinical variables, including urinary incontinence severity, pelvic floor function measures, and oncological risk characteristics, to identify distinct patient phenotypes.”
(See Abstract, Methods)

**Comment 2:**
*“In the results section, which session is meant by improvement in session 3? Is it the last session of the third part of the treatment? Please explain clearly.”*

**Response:**
Thank you for pointing this out. The text has been revised for clarity. We now specify that the third assessment refers to the rehabilitation evaluation conducted after the completion of phase III of the program. The revised sentence reads:

“By the third rehabilitation assessment (conducted after completing phase III of the rehabilitation program), continence rates increased to 88.4%, 77.5%, and 60.0% across the three clusters.”
(See Abstract, Results)

**Main Text**

**Comment 3:**
*“Include the code of ethics in the article.”*

**Response:**
We agree and have expanded the ethics statement to include reference to the Declaration of Helsinki. The revised sentence reads:

“The study was conducted in accordance with the Declaration of Helsinki and approved by the Bioethics Committee of Collegium Medicum, Jan Kochanowski University, Kielce, Poland (approval no. 34/2018). Written informed consent was obtained from all participants.”
(See Methods)

**Comment 4:**
*“Line 97: What was the purpose of ultrasound imaging? This assessment is not mentioned elsewhere in the text.”*

**Response:**
Thank you for highlighting this. We have now specified the purpose of ultrasound imaging in the Methods section. The revised sentence reads:

“Ultrasound sonofeedback was used during all sessions to provide real-time visualization of pelvic floor muscle contractions, enabling patients to learn correct activation and relaxation and to coordinate this activity with breathing.”
(See Methods, Rehabilitation protocol)

**Comment 5:**
*“Were surface electrodes placed on the rectus abdominis and gluteal muscles to check for the absence of accessory muscle contraction? How many channels did the EMG device have, and what was the device model name?”*

**Response:**
These details have now been added. Surface electrodes were placed on the rectus abdominis and right gluteal muscle to prevent accessory muscle activation. We also specified the EMG system used. The text now reads:

“Surface electrodes were placed over the rectus abdominis and right gluteal muscle to monitor and prevent the activation of accessory muscles during pelvic floor contractions. The electromyography system used in this study was a Noraxon Ultium® EMG device equipped with 16 channels, of which four were activated for the purposes of this protocol.”
(See Methods, Rehabilitation protocol)

**Comment 6:**
*“In the exercises section, how were patients able to maintain a 30-second contraction during the first treatment session?”*

**Response:**
We appreciate this question. Patients attempted a 30-second sustained contraction according to the standardized Glazer protocol implemented via Noraxon software. Not all were able to sustain the contraction for the full duration, as this task was used for functional assessment rather than as a therapeutic objective. The sentence has been added:

“While not all patients were able to maintain a full 30-second contraction during the initial session, this step served as a standardized functional assessment rather than a clinical outcome measure in this study.”
(See Methods, Detailed first session procedure)

**Comment 7:**
*“Were the exercises in the second and third phases similar to those in the first phase? How many times per week were the treatment sessions conducted? Please provide a more complete description of the treatment protocol, including how many sessions were in phases two and three and their weekly frequency.”*

**Response:**
Thank you for pointing this out. The text has been expanded to describe the exercise protocol in phases II and III. The revised sentence reads:

“In phases II and III of the rehabilitation program, patients attended five supervised physiotherapy sessions per week. In cases requiring adjunct neuromuscular electrostimulation, a total of 10 sessions were conducted over two weeks, also at a frequency of five sessions per week. The exercise protocol in these phases was similar to that used in phase I and included pelvic floor muscle activation and relaxation in lying, sitting, and standing positions, synchronizing contractions with breathing, and incorporating functional movement patterns.”
(See Methods, Rehabilitation protocol)

**Tables**

**Comment 8:**
*“Some abbreviations in Table 1 are not defined (e.g., PSA). Please verify that all abbreviations are defined in all tables.”*

**Response:**
Thank you for your careful review. We have updated the Abbreviations section under Table 1 to include the definition of PSA as “prostate-specific antigen.”
(See Table 1, Abbreviations)

**Comment 9:**
*“The full name of the ‘ISUP’ is also not provided in the text. Please check and define all abbreviations upon first use.”*

**Response:**
This has been corrected. The full name “International Society of Urological Pathology (ISUP)” is now provided upon first use in the text and included in the Abbreviations list under Table 1.
(See Methods and Table 1, Abbreviations)

We hope that these revisions adequately address all concerns. We are grateful for your valuable feedback, which has helped strengthen the clarity and rigor of our manuscript.

Sincerely,
**Prof. Paweł Macek, PhD, MSc**
Corresponding Author
On behalf of all co-authors
*Collegium Medicum, Jan Kochanowski University of Kielce, Poland*
*Scientific Research, Epidemiology and R&D Centre,*
*Holycross Cancer Centre, Kielce, Poland*
[pawel.macek@onkol.kielce.pl](mailto:pawel.macek@onkol.kielce.pl)

on behalf of all authors
